# Dipeptide IF and Exercise Training Attenuate Hypertension in SHR Rats by Inhibiting Fibrosis and Hypertrophy and Activating AMPKα1, SIRT1, and PGC1α

**DOI:** 10.3390/ijms23158167

**Published:** 2022-07-25

**Authors:** Jou-Hsuan Ho, Rathinasamy Baskaran, Ming-Fu Wang, Zuhair M. Mohammedsaleh, Hong-Siang Yang, Balamuralikrishnan Balasubramanian, Wan-Teng Lin

**Affiliations:** 1 Department of Food Science, Tunghai University, Taichung 407224, Taiwan; jhho@thu.edu.tw (J.-H.H.); g09621013@thu.edu.tw (H.-S.Y.); 2 Department of Bioinformatics and Medical Engineering, Asia University, Taichung 413305, Taiwan; baskaran@asia.edu.tw; 3 Department of Food and Nutrition, Providence University, Taichung 43301, Taiwan; mfwang@pu.edu.tw; 4 Department of Medical Laboratory Technology, Faculty of Applied Medical Sciences, University of Tabuk, Tabuk 71491, Saudi Arabia; zsaleh@ut.edu.sa; 5 Department of Food Science and Biotechnology, College of Life Science, Sejong University, Seoul 05006, Korea; bala.m.k@sejong.ac.kr; 6 Department of Hospitality Management, College of Agriculture, Tunghai University, Taichung 407224, Taiwan

**Keywords:** bioactive peptide, exercise training, fibrosis, hypertrophy, hypertension

## Abstract

Bioactive peptides are physiologically active peptides produced from proteins by gastrointestinal digestion, fermentation, or hydrolysis by proteolytic enzymes. Bioactive peptides are resorbed in their whole form and have a preventive effect against various disease conditions, including hypertension, dyslipidemia, inflammation, and oxidative stress. In recent years, there has been a growing body of evidence showing that physiologically active peptides may have a function in sports nutrition. The present study aimed to evaluate the synergistic effect of dipeptide (IF) from alcalase potato protein hydrolysates and exercise training in hypertensive (SHR) rats. Animals were divided into five groups. Bioactive peptide IF and swimming exercise training normalized the blood pressure and decreased the heart weight. Cardiac, hepatic, and renal functional markers also normalized in SHR rats. The combined administration of IF peptide and exercise offer better protection in SHR rats by downregulating proteins associated with myocardial fibrosis, hypertrophy, and inflammation. Remarkably, peptide treatment alongside exercise activates the PI3K/AKT cell survival pathway in the myocardial tissue of SHR animals. Further, the mitochondrial biogenesis pathway (AMPKα1, SIRT1, and PGC1α) was synergistically activated by the combinatorial treatment of IF and exercise. Exercise training along with IF administration could be a possible approach to alleviating hypertension.

## 1. Introduction

Hypertension is a health problem that affects approximately 1 billion individuals worldwide [1]. During the course of hypertension, increased pressure overload causes changes in heart morphology, notably in the left ventricle (LV), resulting in left ventricular hypertrophy [2]. Hypertrophy develops as a negative feedback loop at first, and contractile function is preserved [3]. When the heart is strained for an extended period of time, cardiac function could decline, leading to heart failure [4].

Mitochondria are the predominant producer of reactive oxygen species (ROS) and the key target of ROS assault during myocardial hypertrophy [5]. Oxidative stress affects heart mitochondria, resulting in impaired mitochondrial activity and severe myocardial hypertrophy [6]. In addition, mitochondrial biogenesis dysfunction is a common pathogenic feature of cardiac hypertrophy [7]. As a result, protecting mitochondrial function and integrity has emerged as an effective therapeutic strategy for treating pathological myocardial hypertrophy [6].

Sirtuin 1 (SIRT1) is an oxidative enzyme that modulates cellular and molecular events such as apoptosis, cell survival, endocrine signaling, chromatin remodeling, and gene transcription [8]. SIRT1 activation promotes mitochondrial biogenesis through its regulatory protein peroxisome proliferator-activated receptor gamma coactivator 1-alpha (PGC-1α), refilling metabolic signaling pathways while inhibiting inflammatory signaling [9]. PGC-1α is a potent transcriptional coactivator of numerous transcription factors and nuclear receptors that regulates metabolic pathways, including oxidative metabolism. PGC-1α is involved in mitochondrial biogenesis and oxidative phosphorylation, which is one of its primary functions. Enhancing PGC-1α expression can improve mitochondrial biogenesis and oxidative phosphorylation in muscle, cardiac, and fat tissue [10,11,12]. A PGC-1α knock-out mice model does not show antioxidant effects following exercise, although exercise training often protects against aging-induced loss of mitochondrial proteins and related cell death [13,14].

Exercise training has several physical and mental health benefits, including preventing obesity, hyperglycemia, hypertension, and hypercholesterolemia [15]. Regular physical activity can enhance physiological function and reduce metabolic problems. Exercise also lowers the risk of aging-related disorders such as hyperglycemia, hypertension, dyslipidemia, cardiovascular disease, and cancer [16,17]. Swimming exercise has been shown to efficiently activate the AMP-activated protein kinase (AMPK)/SIRT1/Forkhead Box O3 (FOXO3) pathway proteins and insulin-like growth factor 1 (IGF1)/phosphoinositide 3-kinases (PI3K)/protein kinase B (Akt) pathway proteins—two important pro-survival signaling pathways. In addition, swimming exercise protects mice against myocardial and liver apoptosis [18,19]. Moderate swimming exercise is a treatment strategy for individuals with hypertension [20]. Reduced exercise tolerance due to hypertension causes structural and functional changes in the heart that deteriorate cardiac function [21]. An approach that integrates exercise training could be a better strategy for alleviating hypertension.

Plant-derived peptides have been studied for their ability to prevent obesity and age-related diseases [22,23]. Potato protein hydrolysate bioactivity was attributed to the intricacy of the generated peptide fragments, including reactive amino acid side chains and hydrophobic regions [24]. The bioactive peptide IF (I-isoleucine; F-phenylalanine) fragment was rich in the alcalase potato protein hydrolysate (APPH) with two aliphatic side chains, which is important for its pharmacological activity [25]. Based on their amino acid residues, bioactive peptides may have antidiabetic action [26].

Myocardial damage and hypertension are linked. Myocardial damage is associated with long-term hypertension. Apoptosis and mitochondrial biogenesis dysfunction in the heart are significantly higher in spontaneously hypertensive rats (SHR) [27]. Therefore, finding therapies to treat hypertension-induced myocardial damage is critical. In SHR rats, angiotensin-converting enzyme (ACE) inhibitors inhibit cardiomyocyte apoptosis [28]. Previous reports have shown that bioactive peptides from soybeans are potent drug candidates for treating hypertension [29,30]. Side effects are one of the major disadvantages of the long-term use of commercial hypertensive drugs. Long peptides are difficult for intestinal cells to absorb and penetrate target cells. Previously, we showed that IF peptides are easily absorbed by the intestine due to their smaller size [31]. As an alternative, in this study, we use active components from functional foods derived from plants to prevent hypertension in SHR. This study examines the molecular mechanisms underlying myocardial damage and the effects of swimming exercise training and IF peptide in spontaneously hypertensive rats.

## 2. Results

### 2.1. Effect of IF Peptide and Exercise Training on Heart Weight, Blood Lipid Profile, Blood Pressure Ejection Fraction, and Fractional Shortening Parameters in Hypertension Rats

**Figure 1:**
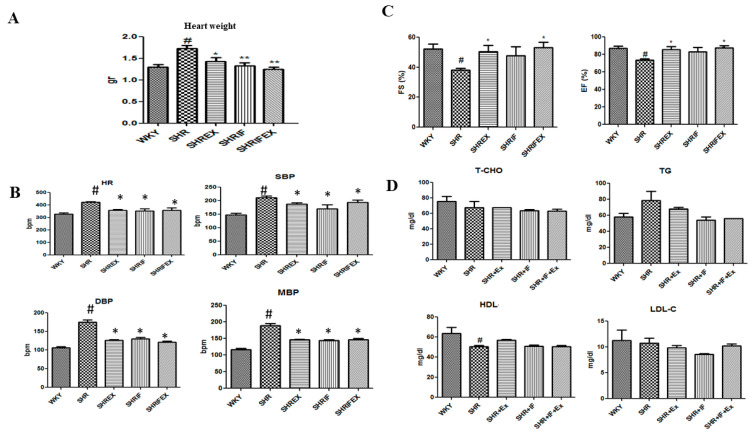
Effect of IF peptide and exercise training on heart weight, blood lipid profile, blood pressure ejection fraction, and fractional shortening parameters in hypertension rats. (**A**) Heart weight (gr, gram). (**B**) Blood parameters such as systolic blood pressure (SBP), diastolic blood pressure (DBP), mean blood pressure (MBP), and heart rate (HR). (**C**) EF, ejection fraction; FS, fractional shortening. (**D**) Blood lipid profile. T-CHO, total cholesterol; TG, triglycerides; HDL, high-density lipoprotein; LDL-C, low-density lipoprotein cholesterol. n = 8, # *p* < 0.01 compared to WKY, * *p* < 0.05, and ** *p* < 0.01 compared to SHR.

The rat heart weights are shown in Figure 1A. The heart weight of the SHR group was significantly higher than control Wistar–Kyoto rats (WKY), which was significantly reduced with IF treatment and exercise training. Systolic blood pressure (SBP), diastolic blood pressure (DBP), mean blood pressure (MBP), and heart rate (HR) were significantly increased in the SHR group compared with the control group. IF administration and exercise training effectively reduced all parameters (Figure 1B). Decreased ejection fraction and fractional shortening in SHR rats were significantly improved after combined treatment of IF and exercise training, whereas IF treatment alone had no effect (Figure 1C). However, there were no significant changes in blood total cholesterol, triglycerides, high-density lipoprotein (HDL), and low-density lipoprotein (LDL) between the animal groups (Figure 1D).

### 2.2. Effect of IF Peptide and Exercise on Heart, Liver, and Kidney Functional Markers

**Figure 2:**
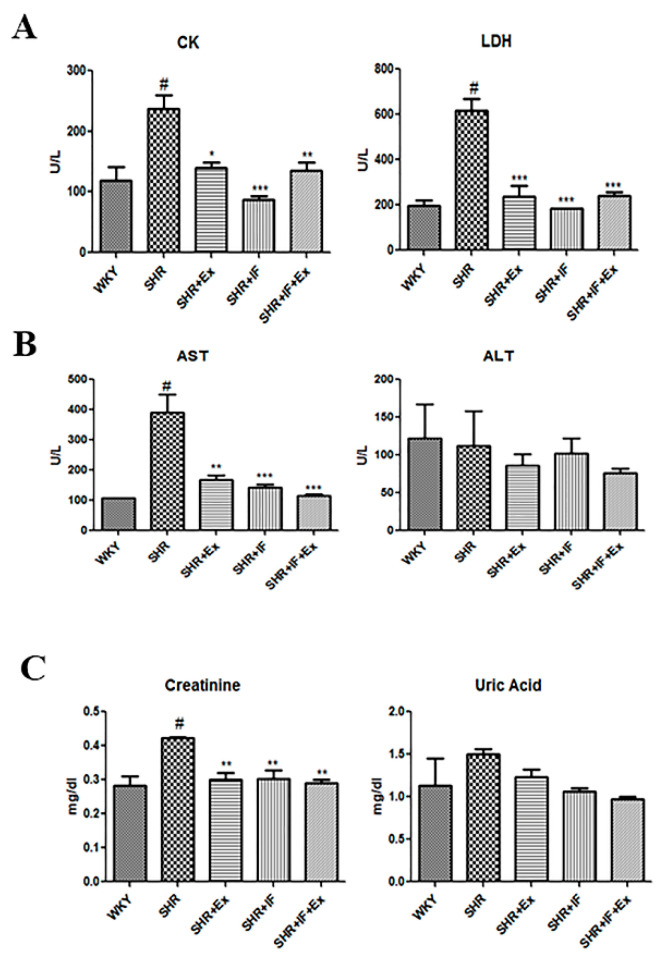
Effect of IF peptide and exercise on heart, liver, and kidney function markers. (**A**) Creatine kinase (CK) and lactate dehydrogenase (LDH). (**B**) Aspartate transaminase (AST) and alanine transaminase (ALT). (**C**) Creatinine and uric acid. n = 8, # *p* < 0.01 compared to WKY, * *p* < 0.05, ** *p* < 0.01, and *** *p* < 0.001 compared to SHR.

Creatine kinase (CK) and lactate dehydrogenase (LDH) are cardiac markers, alanine transaminase (ALT) and aspartate aminotransferase (AST) are hepatic markers, and creatinine and uric acid are renal markers. Heart marker CK and LDH were increased in the SHR animals, which were reduced after IF treatment and exercise training. In liver markers, AST was significantly increased in SHR rats, whereas ALT was not altered significantly. Both exercise and IF treatment significantly reduced AST levels in SHR rats. Kidney marker creatinine was increased in SHR animals and was significantly lower after IF treatment and exercise. Uric acid level was not changed in the SHR group compared to the control group (Figure 2).

### 2.3. Effect of IF Peptide and Exercise Training on Histological Changes in Heart Morphology of Hypertension Rats

**Figure 3:**
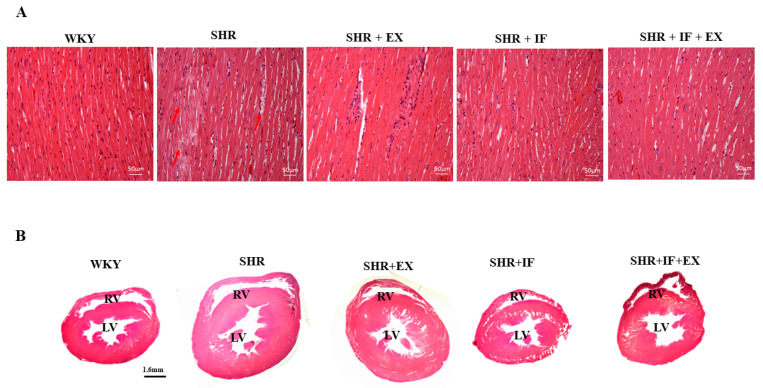
Effect of IF peptide and exercise training on histological changes in heart morphology of hypertension rats. (**A**) HE staining. Red arrow showing histological alterations in SHR rats (**B**) Tomography of cardiac tissues. RV, right ventricle; LV, left ventricle.

Histological changes in the cardiac tissues were evaluated by hematoxylin and eosin (HE) staining (Figure 3A). Severe myofiber disarrangement and deformation of cell structure were observed in the SHR animals, suggesting myocardial damage in SHR rats. However, IF peptide treatment and exercise training in SHR animals reduced histological changes. Tomography of heart tissue sections also showed increased heart volume in SHR rats, which was reduced after IF treatment and exercise (Figure 3B).

### 2.4. IF Treatment and Exercise Prevent Fibrosis in SHR Rats

**Figure 4:**
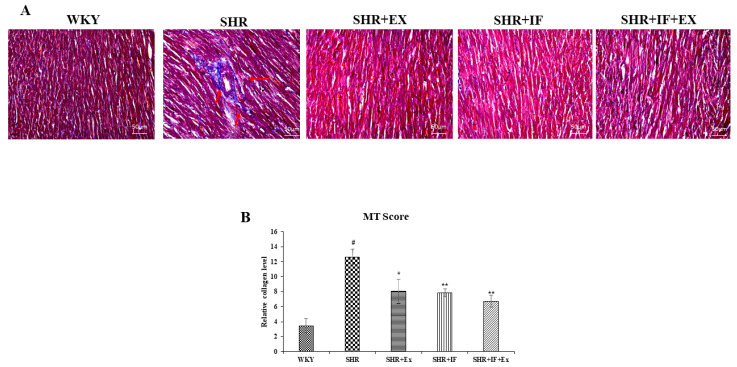
IF treatment and exercise prevent fibrosis in SHR rats. (**A**) Masson’s trichrome staining; Red arrow showing collagen accumulation (**B**) semi-quantitative analysis of fibrosis. MT Score, Masson’s trichrome score. n = 8, # *p* < 0.01 compared to WKY, * *p* < 0.05, and ** *p* < 0.01 compared to SHR.

**Figure 5:**
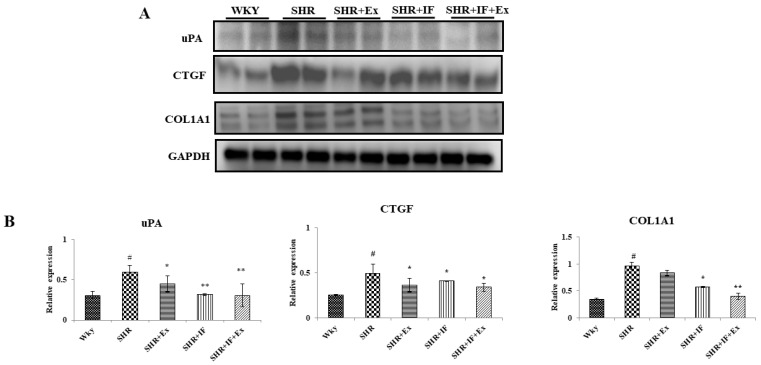
Effects of IF peptide and swimming exercise in cardiac fibrosis pathway. (**A**) Expression of uPA, urokinase-type plasminogen activator protein; CTGF, connective tissue growth factor; COL1A1, collagen type I α 1 chain. (**B**) Densitometric analysis of relative protein expression, normalized with internal control GAPDH. n = 8, # *p* < 0.01 compared to WKY, * *p* < 0.05, and ** *p* < 0.01 compared to SHR.

Masson trichome staining was performed to analyze the collagen accumulation in SHR rats (Figure 4). Masson trichome stains the accumulated collagen in the tissue section in a dark-blue color, which was observed in SHR animals, suggesting the occurrence of fibrosis during hypertension. Both IF treatment and exercise training alone and combined prevented collagen accumulation. Further, we examined the effect of IF treatment and exercise training on the molecular pathway of fibrosis (Figure 5). Urokinase-type plasminogen activator (uPA), connective tissue growth factor (CTGF), and collagen type I, alpha 1 (COL1A1), are the key proteins expressed during fibrosis. Expression of these protein levels was significantly increased in SHR animals compared to the control. On the other hand, IF administration and exercise training significantly reduced the expression of uPA, CTGF, and COL1A1 levels in SHR rats.

### 2.5. Role of IF Peptide and Exercise Training in Alleviating Hypertrophy-Mediated Inflammation in Hypertensive Rats

**Figure 6:**
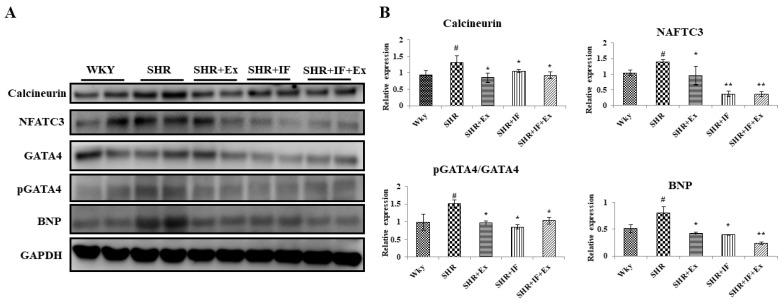
Role of IF peptide and exercise training in alleviating hypertrophy-related protein in hypertensive rats. (**A**) Expression of cardiac hypertrophy-associated markers: calcineurin, nuclear factor of activated T cells 3 (NFATC3), GATA binding protein 4 (GATA4), phosphorylated GATA4 (pGATA4), and brain natriuretic peptide (BNP) proteins. (**B**) Densitometric analysis of relative protein expression, normalized with internal control GAPDH, p-GATA4 is normalized with total GATA4. n = 8, # *p* < 0.01 compared to WKY, * *p* < 0.05, and ** *p* < 0.01 compared to SHR.

**Figure 7:**
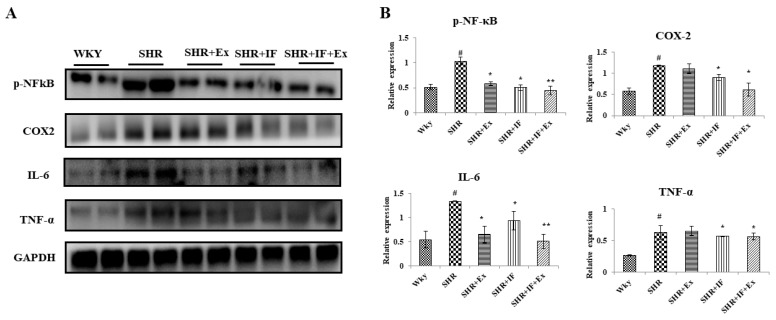
Role of IF peptide and exercise training in alleviating hypertrophy-mediated inflammation in SHR rats. (**A**) Expression of pro-inflammatory signaling protein, nuclear factor kappa-light-chain-enhancer of activated B cells (NF-κB), cyclooxygenase-2 (COX2), and inflammatory cytokines interleukin 6 (IL-6), and tumor necrosis factor alpha (TNF-α). (**B**) Densitometric analysis of relative protein expression, normalized with internal control GAPDH. n = 8, # *p* < 0.01 compared to WKY, * *p* < 0.05, and ** *p* < 0.01 compared to SHR.

From the tomography and heart weight, we found hypertrophy in hypertensive rats. Furthermore, we examined the expression of the hypertrophy pathway proteins calcineurin, nuclear factor of activated T cells 3 (NFATC3), GATA binding protein 4 (GATA4), phosphorylated GATA4 (p-GATA4), and brain natriuretic peptide (BNP) in all the treatment groups (Figure 6). In the SHR group, hypertrophy pathway proteins calcineurin, NFATC3, GATA4, p-GATA4, and BNP were significantly elevated compared to the control. Both IF peptide and exercise training significantly reduced these proteins suggesting an anti-hypertrophy effect. Hypertrophy-mediated inflammation in SHR rats was also analyzed (Figure 7). Nuclear factor kappa-light-chain-enhancer of activated B cells (NF-κB) p65 pro-inflammatory signaling protein was activated, and its downstream regulator cyclooxygenase-2 (COX2) was upregulated in SHR rats. Inflammatory cytokine interleukin 6 (IL-6) and tumor necrosis factor alpha (TNF-α) levels were upregulated in the SHR group suggesting inflammation in SHR rats. However, combined IF treatment and exercise training significantly reduced the NF-κB, COX2, IL-6, and TNF-α.

### 2.6. Effect of IF and Exercise Training on the Cell Survival Pathway in the Heart Tissue of SHR Animals

**Figure 8:**
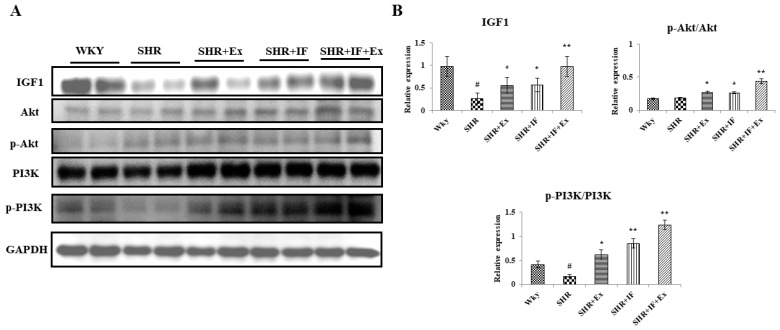
Effect of IF and exercise training on the cell survival pathway in the heart tissue section of SHR animals. (**A**) Expression of survival protein. IGF1, insulin-like growth factor 1; PI3K, phosphoinositide 3-kinases; AKT, protein kinase B. (**B**) Densitometric analysis of relative protein expression, normalized with internal control GAPDH. n = 8, # *p* < 0.01 compared to WKY, * *p* < 0.05, and ** *p* < 0.01 compared to SHR.

The cell survival pathway IGF1/PI3K/Akt plays a crucial role in the heart. We examined the effect of IF and exercise training on the cell survival pathway in SHR rats (Figure 8A). In SHR rats, IGF1 and p-PI3K levels were significantly reduced compared to the control, and p-Akt was not altered. Meanwhile, combined IF treatment and exercise training effectively increased IGF1/PI3K/Akt expression in SHR animals (Figure 8B).

### 2.7. IF Treatment Combined with Exercise Training Alleviates Hypertension through Upregulating AMPKα1, SIRT1, and PGC1α Signaling Pathways

**Figure 9:**
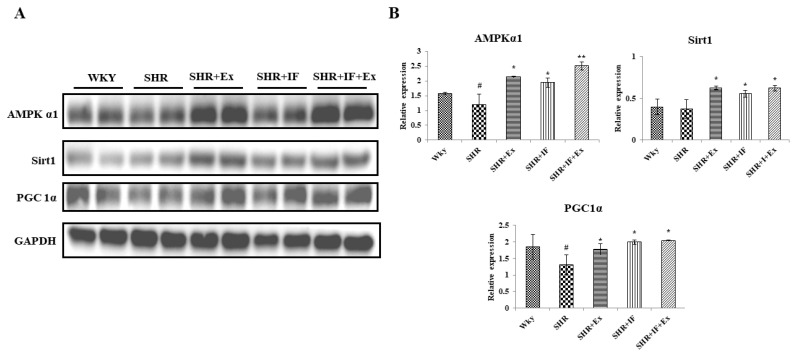
IF treatment combined with exercise training alleviates hypertension by modulating AMPKα1, SIRT1, and PGC1α signaling pathways. (**A**) Expression of AMP-activated protein kinase α1, sirtuin 1, and peroxisome proliferator-activated receptor gamma coactivator 1-alpha proteins. (**B**) Densitometric analysis of relative protein expression, normalized with internal control GAPDH. n = 8, # *p* < 0.01 compared to WKY, * *p* < 0.05 and ** *p* < 0.01 compared to SHR.

We analyzed the mitochondrial biogenesis pathway proteins AMPKα1, SIRT1, and PGC1α in the treatment groups by Western blot analysis (Figure 9). The amount of AMPKα and PGC1α was downregulated in SHR animals, but SIRT1 was not altered. IF treatment and exercise combined and alone significantly upregulated protein levels.

## 3. Discussion

Plant-based compounds or nutraceuticals have been used to treat various medical conditions, including cardiovascular disorders, diabetes, and obesity. Potato protein is high in nutrients and has a well-balanced hydrophilic/hydrophobic amino acid composition, which is crucial for free radical scavenging [32]. The heat treatment used in the potato protein recovery process may impact the solubility and nutritional value of the protein due to inactivation. Enzymatic hydrolysis of potato protein is an alternate method for obtaining functionally active peptides that can solve this problem [33].

The heart is one of the most commonly injured organs in hypertensive patients. Preserving appropriate contractility as blood pressure rises is vital for maintaining heart function and adequate blood flow [34]. The SBP, DBP, MBP, and HR values in the SHR group were considerably greater than those in the WKY group, suggesting the SHR group had higher left ventricular stress, ejection volume, and myocardial dysfunction. Our findings demonstrated that IF therapy combined with swimming exercise might help mitigate cardiac damage by reducing the increased burden on the heart.

Cardiac fibrosis and diastolic dysfunction have been linked to inflammatory responses [35]. Irreversible heart failure is caused by increased extracellular matrix (ECM) thickness caused by exuberant hypertrophy and myocardial fibrosis. Multiple signaling pathways, including tissue-type plasminogen activator protein uPA and CTGF, contribute to orchestrating myocardial fibrosis and atrial fibrillation [36,37]. The current investigation showed that the pro-inflammatory signaling of NF-κB activation and COX2 were significantly upregulated in SHR rats. NF-κB regulates inflammatory cytokine production via the COX2 pathway, which is critical in mediating fibrosis [38,39]. Stress-induced cardiac remodeling (physiological and pathological hypertrophy), permanent functional impairment, and heart failure are the consequences of prolonged cellular adaptation [3]. In this work, we have shown that SHR had higher levels of hypertrophy biomarkers, such as calcineurin, NFATC3, GATA4, and BNP, than control WKY rats. The incidence of pathological myocardial hypertrophy was significantly higher in SHR rats. Hypertrophy is regulated by inflammatory mediators such as TNF-a and IL-6. TNF-α and IL-6 protein levels were considerably higher in SHR rats. In cardiomyocytes, TNF-α influences hypertrophic growth response [40]. NF-κB/COX2 has been implicated in controlling hypertension, inflammation, and I/R damage, all of which can contribute to heart failure [41,42]. IF, a bioactive dipeptide that inhibits uPA-mediated signaling, and swimming exercise effectively decreased fibrotic alterations and prevented SHR-induced fibrosis. Furthermore, it has been shown that blocking uPA or MMP signaling in mice reduces LV fibrosis caused by pressure overload [43].

Physical exercise training reduces cardiomyocyte loss and can be a non-pharmacological treatment for cardiovascular disorders, including hypertension [44]. In hypertensive animals, the PI3K/Akt survival pathway may be stimulated by swimming exercise [19]. Exercise activates the PI3K and Akt signaling pathways, which are crucial regulators in exercise-induced physiological cell proliferation. Additionally, exercise improves cardiac functional and molecular abnormalities by increasing PI3K/Akt expression [45]. In SHR animals, IGF1 and p-PI3K levels were significantly reduced compared to the control, and p-Akt was not altered. Combined IF treatment and exercise training effectively increased the IGF1/PI3K/Akt expression in SHR animals, suggesting a synergistic beneficiary effect in cardiomyocytes. IF treatment and exercise training alone in SHR rats also significantly upregulated the IGF1/PI3K/Akt levels.

Hypertension is linked to increased cellular metabolic activities such as mitochondrial oxidation and the mitochondrial respiratory chain [46]. Increased BP during hypertension-induced changes in the expression levels of longevity proteins such as SIRT1 is due to mitochondrial dysfunction [47]. SIRT1 is a redox-sensitive enzyme that controls cellular death and survival, endocrine signaling, chromatin remodeling, and gene expression [8]. When SIRT1 is activated, PGC-1 is deacetylated and transcriptionally activated, promoting mitochondrial biogenesis. Upregulation of PGC-1α in muscle, heart, and fat tissues promotes mitochondrial biogenesis and oxidative phosphorylation [9,10]. PGC-1α is also required for the antioxidant benefits of exercise training, which protect against hypertension-associated mitochondrial protein deficiencies and cardiomyocyte cell death [48]. SIRT1 is a key mediator that regulates cellular metabolism and inhibits inflammatory signaling [49]. In circumstances such as hypertrophy and myocardial infarction, SIRT1 has been shown to protect cardiomyocytes [50]. SIRT1 is also implicated in lipid metabolism in the heart, oxidative stress in the heart, protection against HFD-induced cardiac inflammation, and glucose intolerance [51]. As a result, therapeutic interventions that increase SIRT1 levels under stressful situations might safeguard the organization and function of myocardial tissue. DIKTNKPVIF potato peptides prevent hypertension by upregulating the mitochondrial biogenesis (AMPKα/SIRT1/PGC1α/p-Foxo3a/Nrf2/CREB) pathway [25]. Another study demonstrated that resveratrol and exercise improve heart function in aging mouse models by activating SIRT1 [52]. Our results align with these reports, where IF peptide and exercise training prevent hypertension by upregulating mitochondrial biogenesis.

According to growing evidence, exercise training offers cardioprotection and restores heart function in various conditions [53,54]. Although exercise training has been shown to reduce the risk of cardiovascular disorders, such as hypertension, each individual’s ability to undertake the requisite intensity and duration of exercise for therapeutic effect varies [55]. As a result, combining a low-to-moderate exercise program with a pharmacological drug might be an effective treatment strategy. Previous research has shown that resveratrol, when used as a therapeutic intervention, improves the cardio-protective benefits of exercise by boosting the SIRT1-related lifespan mechanism [52]. In our present study, IF treatment, along with swimming exercise, prevents hypertension in rats through synergistically modulating AMPKα1, SIRT1, and PGC1α signaling.

In conclusion, our findings show significant evidence for supplementing IF with swimming exercise to reduce hypertension in rats. Our results provided clear in vivo experimental support that a bioactive dipeptide combined with exercise protects the myocardium from hypertension by alleviating fibrosis, hypertrophy, and inflammation through AMPKα1/SIRT1/PGC1α.

## 4. Materials and Methods

### 4.1. Chemicals

All chemicals used in the present study are of analytical grade. Bioactive dipeptide IF (I-isoleucine; F-phenylalanine) was commercially synthesized by DG peptides Co., Ltd., China (Hangzhou, China) and was about 98% purity.

### 4.2. Animals

Twelve-week-old male Wistar–Kyoto (WKY) and SHR rats were purchased from BioLasco Co., Ltd. (Taipei, Taiwan). All the rats were fed a regular commercial rat feed (Laboratory Rodent Diet 5001 purchased from LabDiet, St. Louis, MO, USA), given tap water, and kept at a constant temperature (22 °C) with a 12-h light/dark cycle. Animals were caged as 3 per cage. After 1 week of acclimatization, SHR rats were divided into 4 treatment groups (n = 8), and WKY rats were used as the control. Group I: WKY rats (control); Group II: SHR; Group III: SHR + EX; Group IV: SHR + IF; and Group V: SHR + IF + EX. The treatment lasted around eight weeks. The animals were euthanized after treatment. Finally, the heart tissue and serum were taken and preserved at −80 °C for later analysis.

### 4.3. Treatment Schedule

Bioactive dipeptide IF (I, isoleucine; F, phenylalanine) was purchased commercially from DG peptides Co. Ltd., Hangzhou, China, and was of 98% purity. IF dissolved in PBS at 10 mg/kg BW was administered to the SHR rats for 8 weeks through intragastric injection. The control group received PBS through intragastric injection. For exercise training, animals were allowed to swim freely. Animals were trained to swim for 20, 40, and 60 min in the first, second, and third week, respectively; from the fourth to the eighth week, the animals were allowed to swim for 60 min.

### 4.4. Blood Pressure Measurement

After treatment, heart rate, systolic blood pressure, medium blood pressure, and diastolic blood pressure were measured by the tail-cuff method using a noninvasive blood pressure measurement system (BP-2010 series, Softron, Tokyo, Japan). Before the measurement, the rats were kept in a warm box for 10 min.

### 4.5. Measurement of Blood Serum Biochemical Parameters

The levels of uric acid, creatine, aspartate transaminase, alanine transaminase, and creatine kinase were determined using specific ELISA kits according to the manufacturer’s protocol (Sigma Aldrich, St. Louis, MO, USA).

### 4.6. Echocardiography

Echocardiography was performed for all groups before sacrificing. Rats were anesthetized with isoflurane, and echocardiography was performed using 12 MHz linear transducers and a 5e8 MHz sector transducer (Vivid 3, General Electric Medical Systems Ultrasound, Tirat Carmel, Israel).

### 4.7. Histology Staining

At the end of treatment, a small portion of heart tissue (n = 8) was excised and stored in 10% neutral buffered formalin. Tissue samples were then dehydrated by graded ethanol sequentially and embedded in paraffin wax. For hematoxylin and eosin staining, paraffin-embedded tissue was sectioned serially (about 2 µm thick) and stained with HE dyes. For Masson’s trichrome staining, the tissue sections were stained with Masson’s trichrome dye. Stained tissue sections were documented using a camera-equipped microscope (Carl Zeiss Microscopy, Thornwood, NY, USA).

### 4.8. Western Blot

Heart tissue (100 mg) was washed with phosphate-buffered saline and homogenized with 1 mL of lysis buffer (CelLytic^TM^ MT Cell Lysis Reagent, Sigma-Aldrich, Saint Louis, MO, USA). The samples were centrifuged at 12,000 rpm for 30 min at 4 °C. Supernatant was collected and stored at −80 °C for further experiments. Protein in each sample was quantified by Lowry protein assay; equal amounts of protein (50 μg) were used. Equal amounts of protein were separated in 8–12% gel using an SDS-PAGE apparatus (Bio-Rad Laboratories, Hercules, CA, USA. Separated proteins from the gel were transferred to an ethanol-activated PVDF membrane using a gel transfer unit (Bio-Rad Laboratories, Hercules, CA, USA). Non-fat milk powder (5%) in TBST was used for blocking the PVDF membrane for 1 h at room temperature. After washing thrice with TBST, blots were submerged with a specific primary antibody (1:1000 dilution) in TBST at 4 °C overnight in a shaking rocker. The next day, incubated blots were washed with TBST three times for 5 min each to remove any trace of primary antibody and then incubated with equivalent HRP-conjugated secondary antibody (1:5000 dilution) for 1 h at room temperature in a shaking rocker. After washing the blots thrice with TBST, they were submerged with ECL solutions, and the chemiluminescent signals were detected using a Western blot imaging system (GE Healthcare Life Sciences). GAPDH was used as the internal control.

The antibodies used in the present study and their manufacturer details are as follows: AKT (SC-5298, Santa Cruz Biotechnology, Santa Cruz, CA, USA), p-AKT (no. 9275s, Cell Signaling, Baltimore, MD, USA), AMPKα1 (SC-19128, Santa Cruz Biotechnology, Santa Cruz, CA, USA), BNP (SC-18818, Santa Cruz Biotechnology, Santa Cruz, CA, USA), Calcineurin (610259, BD Biosciences, San Jose, CA, USA), COL1A1 (SC-28657, Santa Cruz Biotechnology, Santa Cruz, CA, USA), CTGF (SC-14939, Santa Cruz Biotechnology, Santa Cruz, CA, USA), COX2 (SC-1745, Santa Cruz Biotechnology, Santa Cruz, CA, USA), GAPDH (SC-25778, Santa Cruz Biotechnology, Santa Cruz, CA, USA), GATA4 (SC-25310, Santa Cruz Biotechnology, Santa Cruz, CA, USA), p-GATA4 (SC-32823, Santa Cruz Biotechnology, Santa Cruz, CA, USA), IGF1 (SC-9013, Santa Cruz Biotechnology, Santa Cruz, CA, USA), IL-6 (SC-7920, Santa Cruz Biotechnology, Santa Cruz, CA, USA), NFATC3 (SC-1152, Santa Cruz Biotechnology, Santa Cruz, CA, USA), p-NF-κB (#3033L, Cell Signaling, Baltimore, MD, USA), PGC-1α (SC-13067, Santa Cruz Biotechnology, Santa Cruz, CA, USA), PI3K (SC-423, Santa Cruz Biotechnology, Santa Cruz, CA, USA), p-PI3K (SC-12929, Santa Cruz Biotechnology, Santa Cruz, CA, USA), SIRT1 (SC-15404, Santa Cruz Biotechnology, Santa Cruz, CA, USA), TNF-α (SC-1350, Santa Cruz Biotechnology, Santa Cruz, CA, USA), and uPA (SC-14019, Santa Cruz Biotechnology, Santa Cruz, CA, USA).

### 4.9. Statistical Analysis

The data were analyzed by SPSS 18.0 software, and one-way analysis of variance (one-way ANOVA) and Tukey’s multiple comparisons test was used to compare the statistical difference between the treatment groups. Experimental data are expressed as mean ± SD. *p* < 0.05 indicates a significant difference, and *p* < 0.01 indicates an extremely significant difference.

## Data Availability

The data that support the findings of this study are available on request from the corresponding author.

## References

[1] Egan B.M., Kjeldsen S.E., Grassi G., Esler M., Mancia G. (2019). The global burden of hypertension exceeds 1.4 billion people: Should a systolic blood pressure target below 130 become the universal standard?. J. Hypertens..

[2] Jekell A., Nilsson P.M., Kahan T. (2018). Treatment of hypertensive left ventricular hypertrophy. Curr. Pharm. Des..

[3] Nakamura M., Sadoshima J. (2018). Mechanisms of physiological and pathological cardiac hypertrophy. Nat. Rev. Cardiol..

[4] Yildiz M., Oktay A.A., Stewart M.H., Milani R.V., Ventura H.O., Lavie C.J. (2020). Left ventricular hypertrophy and hypertension. Prog. Cardiovasc. Dis..

[5] Ma M., Chen W., Hua Y., Jia H., Song Y., Wang Y. (2021). Aerobic exercise ameliorates cardiac hypertrophy by regulating mitochondrial quality control and endoplasmic reticulum stress through M2AChR. J. Cell. Physiol..

[6] Sawyer D.B., Siwik D.A., Xiao L., Pimentel D.R., Singh K., Colucci W.S. (2002). Role of oxidative stress in myocardial hypertrophy and failure. J. Mol. Cell. Cardiol..

[7] Karamanlidis G., Bautista-Hernandez V., Fynn-Thompson F., Del Nido P., Tian R. (2011). Impaired mitochondrial biogenesis precedes heart failure in right ventricular hypertrophy in congenital heart disease. Circ. Heart Fail..

[8] Anastasiou D., Krek W. (2006). SIRT1: Linking adaptive cellular responses to aging-associated changes in organismal physiology. Physiology.

[9] Higashida K., Kim S.H., Jung S.R., Asaka M., Holloszy J.O., Han D.-H. (2013). Effects of resveratrol and SIRT1 on PGC-1α activity and mitochondrial biogenesis: A reevaluation. PLoS Biol..

[10] Fernandez-Marcos P.J., Auwerx J. (2011). Regulation of PGC-1α, a nodal regulator of mitochondrial biogenesis. Am. J. Clin. Nutr..

[11] Gureev A.P., Shaforostova E.A., Popov V.N. (2019). Regulation of mitochondrial biogenesis as a way for active longevity: Interaction between the Nrf2 and PGC-1α signaling pathways. Front. Genet..

[12] Jia D., Hou L., Lv Y., Xi L., Tian Z. (2019). Postinfarction exercise training alleviates cardiac dysfunction and adverse remodeling via mitochondrial biogenesis and SIRT1/PGC-1α/PI3K/Akt signaling. J. Cell. Physiol..

[13] Chinsomboon J., Ruas J., Gupta R.K., Thom R., Shoag J., Rowe G.C., Sawada N., Raghuram S., Arany Z. (2009). The transcriptional coactivator PGC-1α mediates exercise-induced angiogenesis in skeletal muscle. Proc. Natl. Acad. Sci. USA.

[14] Handschin C., Chin S., Li P., Liu F., Maratos-Flier E., LeBrasseur N.K., Yan Z., Spiegelman B.M. (2007). Skeletal muscle fiber-type switching, exercise intolerance, and myopathy in PGC-1α muscle-specific knock-out animals. J. Biol. Chem..

[15] Wu N., Bredin S.S., Guan Y., Dickinson K., Kim D.D., Chua Z., Kaufman K., Warburton D.E. (2019). Cardiovascular health benefits of exercise training in persons living with type 1 diabetes: A systematic review and meta-analysis. J. Clin. Med..

[16] Pagano G., Pallardó F.V., Lyakhovich A., Tiano L., Fittipaldi M.R., Toscanesi M., Trifuoggi M. (2020). Aging-related disorders and mitochondrial dysfunction: A critical review for prospect mitoprotective strategies based on mitochondrial nutrient mixtures. Int. J. Mol. Sci..

[17] Canfield C.-A., Bradshaw P.C. (2019). Amino acids in the regulation of aging and aging-related diseases. Transl. Med. Aging.

[18] Thomson D.M. (2018). The role of AMPK in the regulation of skeletal muscle size, hypertrophy, and regeneration. Int. J. Mol. Sci..

[19] Lin J.-Y., Kuo W.-W., Baskaran R., Kuo C.-H., Chen Y.-A., Chen W.S.-T., Ho T.-J., Day C.H., Mahalakshmi B., Huang C.-Y. (2020). Swimming exercise stimulates IGF1/PI3K/Akt and AMPK/SIRT1/PGC1α survival signaling to suppress apoptosis and inflammation in aging hippocampus. Aging.

[20] Tanaka H., Bassett Jr D.R., Howley E.T., Thompson D.L., Ashraf M., Rawson F.L. (1997). Swimming training lowers the resting blood pressure in individuals with hypertension. J. Hypertens..

[21] Kokkinos P.F., Papademetriou V. (2000). Exercise and hypertension. Coron. Artery Dis..

[22] Katayama S., Corpuz H.M., Nakamura S. (2021). Potential of plant-derived peptides for the improvement of memory and cognitive function. Peptides.

[23] Lai P.-F., Baskaran R., Kuo C.-H., Day C.H., Chen R.-J., Ho T.-J., Yeh Y.-L., Padma V.V., Lai C.-H., Huang C.-Y. (2021). Bioactive dipeptide from potato protein hydrolysate combined with swimming exercise prevents high fat diet induced hepatocyte apoptosis by activating PI3K/Akt in SAMP8 mouse. Mol. Biol. Rep..

[24] Udenigwe C.C., Udechukwu M.C., Yiridoe C., Gibson A., Gong M. (2016). Antioxidant mechanism of potato protein hydrolysates against in vitro oxidation of reduced glutathione. J. Funct. Foods.

[25] Lin W.T., Nithiyanantham S., Hsieh D.J.Y., Chen R.J., Day C.H., Liao J.Y., Kuo C.H., Mahalakshmi B., Kuo W.W., Huang C.Y. (2020). Bioactive peptides attenuate cardiac apoptosis in spontaneously hypertensive rat hearts through activation of autophagy and mitochondrial biogenesis pathway. Environ. Toxicol..

[26] Marthandam Asokan S., Wang T., Su W.-T., Lin W.-T. (2019). Antidiabetic effects of a short peptide of potato protein hydrolysate in STZ-induced diabetic mice. Nutrients.

[27] Tang Y., Mi C., Liu J., Gao F., Long J. (2014). Compromised mitochondrial remodeling in compensatory hypertrophied myocardium of spontaneously hypertensive rat. Cardiovasc. Pathol..

[28] Zhu Y.-C., Zhu Y.-Z., Gohlke P., Stauss H.M., Unger T. (1997). Effects of angiotensin-converting enzyme inhibition and angiotensin II AT1 receptor antagonism on cardiac parameters in left ventricular hypertrophy. Am. J. Cardiol..

[29] Li T., Zhang X., Ren Y., Zeng Y., Huang Q., Wang C. (2022). Antihypertensive effect of soybean bioactive peptides: A review. Curr. Opin. Pharmacol..

[30] Chatterjee C., Gleddie S., Xiao C.-W. (2018). Soybean bioactive peptides and their functional properties. Nutrients.

[31] Asokan S.M., Wang T., Wang M.-F., Lin W.-T. (2020). A novel dipeptide from potato protein hydrolysate augments the effects of exercise training against high-fat diet-induced damages in senescence-accelerated mouse-prone 8 by boosting pAMPK/SIRT1/PGC-1α/pFOXO3 pathway. Aging.

[32] Rahimi R., Gavlighi H.A., Sarteshnizi R.A., Barzegar M., Udenigwe C.C. (2022). In vitro antioxidant activity and antidiabetic effect of fractionated potato protein hydrolysate via ultrafiltration and adsorption chromatography. LWT.

[33] Chiang W.-D., Shih C.-J., Chu Y.-H. (1999). Functional properties of soy protein hydrolysate produced from a continuous membrane reactor system. Food Chem..

[34] Schwingshackl L., Chaimani A., Schwedhelm C., Toledo E., Pünsch M., Hoffmann G., Boeing H. (2019). Comparative effects of different dietary approaches on blood pressure in hypertensive and pre-hypertensive patients: A systematic review and network meta-analysis. Crit. Rev. Food Sci. Nutr..

[35] Chen X., Qian J., Wang L., Li J., Zhao Y., Han J., Khan Z., Chen X., Wang J., Liang G. (2018). Kaempferol attenuates hyperglycemia-induced cardiac injuries by inhibiting inflammatory responses and oxidative stress. Endocrine.

[36] Huang C.Y., Nithiyanantham S., Liao J.Y., Lin W.T. (2020). Bioactive peptides attenuate cardiac hypertrophy and fibrosis in spontaneously hypertensive rat hearts. J. Food Drug Anal..

[37] Lin Y.-C., Lin Y.-C., Kuo W.-W., Shen C.-Y., Cheng Y.-C., Lin Y.-M., Chang R.-L., Padma V.V., Huang C.-Y., Huang C.-Y. (2018). Platycodin D reverses pathological cardiac hypertrophy and fibrosis in spontaneously hypertensive rats. Am. J. Chin. Med..

[38] Shehzad A., Rehmat S., Ul-Islam S., Ahmad R., Aljafary M., Alrushaid N.A., Al-Suhaimi E.A. (2020). Lirioresinol B dimethyl ether inhibits NF-κB and COX-2 and activates IκBα expression in CCl4-induced hepatic fibrosis. BMC Complementary Med. Ther..

[39] Wei J., Deng X., Li Y., Li R., Yang Z., Li X., Song S., Shi Y., Duan H., Wu H. (2021). PP2 Ameliorates Renal Fibrosis by Regulating the NF-κB/COX-2 and PPARγ/UCP2 Pathway in Diabetic Mice. Oxidative Med. Cell. Longev..

[40] Rolski F., Błyszczuk P. (2020). Complexity of TNF-α signaling in heart disease. J. Clin. Med..

[41] Amirshahrokhi K., Zohouri A. (2021). Carvedilol prevents pancreatic β-cell damage and the development of type 1 diabetes in mice by the inhibition of proinflammatory cytokines, NF-κB, COX-2, iNOS and oxidative stress. Cytokine.

[42] Lin Y.-Y., Hong Y., Zhou M.-C., Huang H.-L., Shyu W.-C., Chen J.-S., Ting H., Cheng Y.-J., Yang A.-L., Lee S.-D. (2020). Exercise training attenuates cardiac inflammation and fibrosis in hypertensive ovariectomized rats. J. Appl. Physiol..

[43] Heymans S., Lupu F., Terclavers S., Vanwetswinkel B., Herbert J.-M., Baker A., Collen D., Carmeliet P., Moons L. (2005). Loss or Inhibition of uPA or MMP-9 Attenuates LV Remodeling and Dysfunction after Acute Pressure Overload in Mice. Am. J. Pathol..

[44] Soares L., Drummond F., Lavorato V., Carneiro-Junior M., Natali A. (2018). Exercise training and pulmonary arterial hypertension: A review of the cardiac benefits. Sci. Sports.

[45] Palabiyik O., Tastekin E., Doganlar Z.B., Tayfur P., Dogan A., Vardar S.A. (2019). Alteration in cardiac PI3K/Akt/mTOR and ERK signaling pathways with the use of growth hormone and swimming, and the roles of miR21 and miR133. Biomed. Rep..

[46] Chan S.H., Wu K.L., Chang A.Y., Tai M.-H., Chan J.Y. (2009). Oxidative impairment of mitochondrial electron transport chain complexes in rostral ventrolateral medulla contributes to neurogenic hypertension. Hypertension.

[47] Puddu P., Puddu G.M., Cravero E., De Pascalis S., Muscari A. (2007). The putative role of mitochondrial dysfunction in hypertension. Clin. Exp. Hypertens..

[48] Mata M., Sarrion I., Milian L., Juan G., Ramon M., Naufal D., Gil J., Ridocci F., Fabregat-Andres O., Cortijo J. (2012). PGC-1α induction in pulmonary arterial hypertension. Oxidative Med. Cell. Longev..

[49] Zhang J., Ren D., Fedorova J., He Z., Li J. (2020). SIRT1/SIRT3 modulates redox homeostasis during ischemia/reperfusion in the aging heart. Antioxidants.

[50] Hajializadeh Z., Khaksari M. (2021). The protective effects of 17-β estradiol and SIRT1 against cardiac hypertrophy: A review. Heart Fail. Rev..

[51] Cheng B., Gao W., Wu X., Zheng M., Yu Y., Song C., Miao W., Yang Z., He Y., Liu C. (2020). Ginsenoside Rg2 ameliorates high-fat diet-induced metabolic disease through SIRT1. J. Agric. Food Chem..

[52] Lin C.-H., Lin C.-C., Ting W.-J., Pai P.-Y., Kuo C.-H., Ho T.-J., Kuo W.-W., Chang C.-H., Huang C.-Y., Lin W.-T. (2014). Resveratrol enhanced FOXO3 phosphorylation via synergetic activation of SIRT1 and PI3K/Akt signaling to improve the effects of exercise in elderly rat hearts. Age.

[53] Powers S.K., Lennon S.L., Quindry J., Mehta J.L. (2002). Exercise and cardioprotection. Curr. Opin. Cardiol..

[54] Thijssen D.H., Redington A., George K.P., Hopman M.T., Jones H. (2018). Association of exercise preconditioning with immediate cardioprotection: A review. JAMA Cardiol..

[55] Improta Caria A.C., Nonaka C.K.V., Pereira C.S., Soares M.B.P., Macambira S.G., Souza B.S.d.F. (2018). Exercise training-induced changes in microRNAs: Beneficial regulatory effects in hypertension, type 2 diabetes, and obesity. Int. J. Mol. Sci..

